# Moderate dietary weight loss reduces myocardial triglycerides in obese women

**DOI:** 10.1186/1532-429X-14-S1-P69

**Published:** 2012-02-01

**Authors:** Wolfgang Utz, Sven Haufe, Stefan Engeli, Martin Pofahl, Julius Traber, Friedrich Luft, Jens Jordan, Jeanette Schulz-Menger

**Affiliations:** 1 Working Group Cardiac MR Medical Faculty, Charité Campus Buch and HELIOS Klinikum Berlin Buch, Berlin, Germany; 2 Institute of Clinical Pharmacology, Hannover Medical School, Hannover, Germany; 3 Experimental and Clinical Research Center, Charité Medical School and Max Delbrück Center for Molecular Medicine, Berlin, Germany

## Background

Excessive myocardial triglyceride (MTG) content in obesity and type 2 diabetes is associated with impaired cardiac function. Previous studies suggest that MTG could be mobilized through lifestyle interventions. We assessed influences of moderate dietary weight loss in non diabetic obese women on MTG content and cardiac function.

## Methods

Non diabetic overweight and obese women were submitted to a six months hypocaloric diet with either fat or carbohydrate restriction. Cardiac structure and function was assessed by magnetic resonance imaging (MRI) and MTG content by proton spectroscopy (MRS) in 35 subjects at baseline and follow-up. Anthropometric and metabolic parameters as well as cardio-respiratory fitness were measured.

## Results

**Figure 1:**
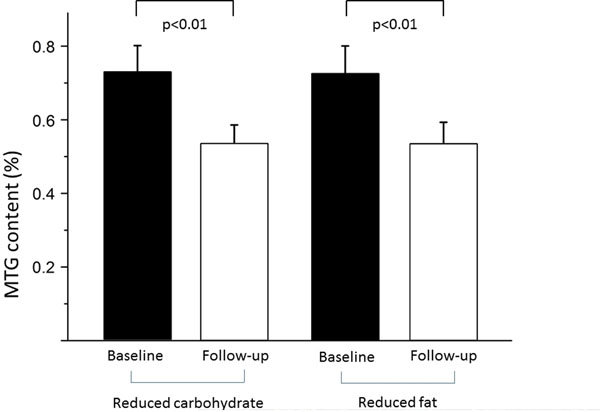
Myocardial triglyceride content before and after weight loss.

**Figure 2:**
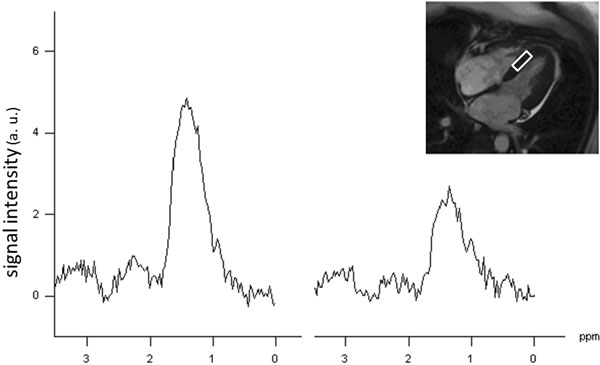
Lipid spectra normalized to water signal in one subject before (left) and after (right) intervention showing reduced MTG at FU (inlet depicts voxel position).

An average weight reduction of 6.0±3.7 kg at six month was associated with a relative decrease of MTG of 26% (from 0.73±0.30% at baseline to 0.54±0.23% at follow-up, p<0.001, Figure 1). The response was similar with carbohydrate and fat restriction. Lipid spectrum before (left) and after (right) weight loss in one subject is shown in Figure 2. Diastolic function expressed as ratio of peak filling rate in E- and A-Phase (PFRE/PFRA) was unchanged. Reductions of left atrial size (21.5±3.3cm2 to 19.8±3.1cm2, p=0.002), the ratio of PFRE and left ventricular lengthening velocity PLV (8.1±2.4 to 7.3±2.3, p<0.001) and fat free mass (55.0±7.1kg to 52.5±6.7kg, p=0.007) reflected altered loading conditions after diet, but did not correlate to MTG content.

## Conclusions

Moderate dietary weight loss significantly reduced MTG content in women with uncomplicated obesity. Macronutrient composition of the diet did not significantly affect the extent of MTG reduction.

## Funding

None.

